# Biodegradation assessment tests of biopolymers in standardised water: different sources of variability

**DOI:** 10.1007/s10532-025-10143-3

**Published:** 2025-05-17

**Authors:** David Gutiérrez-Rial, Iria Villar, Pilar Feijóo, Benedicto Soto, Josefina Garrido, Salustiano Mato

**Affiliations:** 1 Departamento de Ecoloxía e Bioloxía Animal, Facultade de Bioloxía, Universidade de Vigo, Campus Lagoas Marcosende s/n 36310, Vigo, España; 2 Departamento de Bioloxía Vexetal e Ciencias do Solo, Universidade de Vigo, Campus Lagoas Marcosende s/n 36310, Vigo, España

**Keywords:** Polyhydroxyalkanoates, Polylactic acid, Biopolymers, Pollution, Sustainability, Circular economy

## Abstract

This study assessed the ultimate biodegradation degree of two resins, polyhydroxybutyrate and polylactic acid (PHB and PLA), and three commercial biobased bags (BMAT, BGREEN, and BBEIGE) through the measurement of oxygen consumption in closed respirometers. Activated sludge from a wastewater treatment plant (WWTP) was used as the inoculum, cellulose was used as the reference material, and five trials were conducted with two different devices under identical conditions, with a 28-day incubation period. The results revealed statistically significant differences in the biochemical oxygen demand (BOD) measurements for cellulose, PHB, and PLA between the two devices and within the same devices across different trials. The degree of biodegradation (D_t_), calculated as the percentage of theoretical oxygen demand (ThOD), varied depending on the device and trial. For cellulose, D_t_ ranged from 61 to 93%; for PLA, the maximum Dt was 6%; and for PHB, D_t_ oscillated between 16 and 72%. These findings highlight the critical importance of carefully selecting the testing equipment, as it significantly influences biodegradation results, in addition to the already known interlaboratory variability caused by the inoculum.

## Introduction

Plastics have become one of the products in greatest demand by society. Owing to their low production cost and versatility, a world without plastics seems unimaginable (Geyer et al. 2017). Some plastics can be reused or recycled, but most must be discarded after their first use (Azevedo-Santos et al. 2021). Moreover, conventional oil-based polymers are not biodegradable under environmental conditions once they accumulate in natural ecosystems (Shen et al. 2020). Traditionally, much concern has been raised about plastic pollution in oceans, but currently, plastic is known to be present in all environments (Williams and Rangel-Buitrago 2022).

In this context, freshwater ecosystems represent one of the largest sinks for plastic waste, and rivers constitute one of the most important pathways through which plastic waste reaches the ocean. Each year, rivers release more than 2.7 million tons (m.t.) of plastic into coasts worldwide (Lebreton et al. 2018; Meijer et al. 2021). Nevertheless, the assumption that all plastics that reach rivers end up in the ocean is not completely true. A large fraction of plastic is not found back afloat in the ocean and is considered lost (Ter Halle et al. 2016). Hence, the fate of this missing fraction of plastic waste remains unknown, but several authors indicate that it might accumulate in ocean or riverine sediments or on shorelines (Taylor et al. 2016).

Conventional plastics in river systems are subjected to degradation processes, mainly mechanical degradation through physical contact with other objects, but can also be affected by thermal and UV degradation or photodegradation. However, biodegradation mechanisms in aquatic environments do not contribute significantly to the degradation of oil-based polymers because of the slow pace of this process (Bergmann et al. 2015). In recent decades, the development of biodegradable polymers has emerged as an optimal alternative to combat the global plastic waste problem (Manavitehrani et al. 2016; Tassinari et al. 2023). With increasing awareness of plastic pollution, the need for degradable plastics has increased. Today, compostable starch blends, such as polylactic acid (PLA), polybutylene adipate terephthalate (PBAT) or polyhydroxyalkanoate (PHA), are produced in tons at the industrial scale (Schneiderman and Hillmyer 2017). However, in most cases, the term biodegradable cannot be interpreted literally, as most standardised tests are performed under optimal conditions, resulting in a higher degree of ultimate biodegradation. Therefore, biodegradation in natural ecosystems usually does not occur or is very slow compared with results obtained in the laboratory, where conditions are optimal for the process (Lambert and Wagner 2017).

The biodegradation process is caused by the activity of bacterial and fungal enzymes and can be defined as the mineralisation of organic matter leading to CO_2_ and H_2_O under aerobic conditions and an increase in the biomass of microorganisms (Haider et al. 2019; Bacha et al. 2023). The first guidelines for testing the ultimate degree of biodegradation of plastics were given by the International Standards Organization (ISO), and at this time, the basis regarding biodegradability in various environments (soil, sea water and freshwater) depends on it. A variety of tests exist, each with specified conditions. These standardised tests can range from a period of less than two months (ISO 2019) to a maximum of twenty-four months (Haider et al. 2019), depending on the environment, but a minimum incubation period is not specified. Moreover, these methods are usually developed at temperatures higher than those in real natural environments (Filiciotto and Rothenberg 2021). The standardisation of these tests was a significant advancement in the field of plastics. However, the methods used to analyse the biodegradation of plastics have historically been subjected to high variability across laboratories (Muller et al. 1992; Chiellini et al. 1999; Mezzanotte et al. 2005; Krzan et al. 2006).

Therefore, the aim of this work was to assess the sources of variability affecting the international standard ISO 14851 for the determination of the ultimate aerobic biodegradability of plastic materials in an aqueous medium. For this purpose, the biodegradability of some of the most produced biobased and biodegradable bioplastics was assessed: Two resins (PHB and PLA) and three different biobased bags. The specific objectives were to identify differences in biodegradation test results attributable to the use of different devices and the effect of inoculum source reproducibility. Thus, this study aims to provide clarity regarding potential biases in current water biodegradation testing standards, thereby enhancing the methodology and reducing the risk of incorrectly labeling non-biodegradable compounds as biodegradable.

## Materials and methods

The biodegradation of the selected materials was evaluated by measuring the oxygen consumption in closed respirometers in an aqueous medium under aerobic conditions. The tests were carried out following the modifications of the standard established by López-Ibáñez and Beiras (2022), who proposed a reduction in the incubation time to 28 days in marine test standards for test materials with particle sizes lower than 250 µm.

The results are expressed as biochemical oxygen demand (BOD_28_) and were calculated according to Eq. 1.

1 $$ BOD_{28} = \frac{{BOD_{t} - BOD_{Bt} }}{{\rho_{TC} }} \times 100 $$

where BOD_28_ is the amount of oxygen consumed (mg g^−1^) for each of the treatments after the incubation period. BOD_t_ is the oxygen consumed in flasks with test material, BOD_BT_ is the oxygen consumed in the blanks (both in mg L^−1^), and *ρ*_TC_ is the concentration of the tested material in the respirometer expressed in g L^−1^. Next, biodegradation (D_t_ expressed in %) was calculated as the ratio of the BOD_28_ to its theoretical oxygen demand (ThOD).

2 $$ D_{t} = \frac{{BOD_{28} }}{ThOD} \times 100 $$

where D_t_ is the biodegradation expressed as a percentage of ThOD, BOD_28_ is calculated with Eq. 1 for each test material, and ThOD is the theoretical oxygen demand of each tested polymer (Table 1). The test materials were classified as poorly biodegradable (D_t_ < 20% ThOD), potentially biodegradable (20–60% ThOD) or readily biodegradable (D_t_ > 60% ThOD). This is the same criterion used by López Ibáñez and Beiras (2022), who adapted the benchmarks established by OSPAR (2015) to refer to the persistence of chemicals.

**Table 1:** Characteristics of the different test materials used in the biodegradation tests

Code	Type	Materials ^a^	Manufacturer	Form	Size (µm)	ThOD (mg/g)
Cellulose	Commercial	Cellulose TLC grade	Sigma–Aldrich	Micronised	< 250	1184
PLA	Resin	Polylactic acid resin	NaturePlast	Micronised	< 250	1655
PHB	Resin	Polyhydroxybutirate resin	Helian Polymers	Micronised	< 250	1636
BGREEN	Commercial	PBAT + PLA + Corn starch	Green Maker	Micronised	< 250	1716
BMAT	Commercial	Corn starch + biodegradable polymers	BioBag	Micronised	< 250	1464
BBEIGE	Commercial	Corn starch	EcoPack	Micronised	< 250	3447

*PBAT*: Polybutylene Adipate Terephthalate, *PHB*: Polyhydroxybutyrate, *PLA*: Polylactic acid

^a^Material polymer: Composition of the products according to the manufacturer’s descriptions

### Test materials

A total of 5 test materials, 2 resins and 3 commercial bags, were employed (Table 1). PHB and PLA resins were selected due to the fact they are among the most produced biobased and biodegradable polymers worldwide. Commercial bioplastic bags were selected because they are already available on the market, allowing for a practical approach to assessing a real-life environmental issue in this study. All materials were previously micronised via a ZM200 ultracentrifuge mill (Restch, Verder Scientific) and sieved through a 250 µm metallic mesh. Microcrystalline TLC grade cellulose, a fully biodegradable compound, was used as a reference material. An elemental analysis was carried out on each material to determine the carbon (C), hydrogen (H), chlorine (Cl), nitrogen (N), sulfur (S), phosphorus (P), sodium (Na) and oxygen (O) contents to subsequently calculate the ThOD of each material.

### Test solution and microbial inoculum

To simulate a natural environment, standard test media were prepared using analytical grade reagents: KH_2_PO_4_ (Labkem), K_2_HPO_4_ (Panreac), Na_2_HPO_4_·2H_2_O (Labkem), NH_4_Cl (Panreac), MgSO_4_·7H_2_O (Labkem), CaCl_2_·2H_2_O (Labkem), and FeCl_3_·6H_2_O (Labkem). The medium was prepared according to the international standard 14851 (ISO 2019). The inoculum used was activated sludge from the Guillarei Wastewater Treatment Plant (WWTP) (Tui, Galicia, Spain). The sludge was sampled directly from the clarifier tank and transported to the laboratory for characterisation (Table 2). The number of colony forming units (CFUs) was determined via the pour-plate method according to ISO 6222. Moreover, elemental and nutrient analyses were carried out at the Scientific and Technological Support Centre for Research of the University of Vigo. Anions (Cl^–^, NO_2_^–^, NO_3_^–^, PO_4_^3−^, and SO_4_^2−^) and ammonium (NH_4_^+^) were determined by ionic chromatography (Metrohm 940 IC) and colorimetry (Spectroquant Nova 60), respectively. The total organic carbon (TOC), total carbon (TC) and total nitrogen (TN) contents were determined via thermocatalytic decomposition (Analytik Jena multi N/C 3100).

**Table 2:** Characteristics of the activated sludge from the WWTP used as inoculum

	CFU (nº/ml)	SS	TOC	TC	TN	Cl	NO^2^	NO^3^	PO^4^	SO^4^	NH_4_^+^
Inoculum	10^5^	2230	58.8	84.5	15.4	266	0.08	1.6	92.3	23.2	2.2

CFU: colony formation units (n° ml ^−1^); SS: suspended solids (mg L^−1^) TOC: total organic carbon (mg L^−1^); TC: total carbon (mg L^−1^); TN: total nitrogen (mg L^−1^); Cl: chloride (mg L^−1^); NO_2_: nitrite (mg L^−1^); NO_3_: nitrate (mg L^−1^); PO_4_: phosphate (mg L^−1^); SO_4_: sulfate (mg L^−1^); and NH_4_^+^: ammonia (mg L^−1^)

The inoculum was mixed with the standard test medium to create the test mixture. First, the activated sludge was shaken in an orbital rotator for 2 min, and after 30 min, the decantation supernatant was extracted. Second, the supernatant was added to the nutrient mixture at a proportion of 5% volume. The pH was determined to be 7 ± 0.1, and the number of CFUs in the test mixture was 10^5^, within the range accepted by ISO 14851.

### Equipment

To develop this work, two different laboratory instruments were used, with some differences between them. On the one hand, twelve OxiTop®-C measuring heads with an inductive stirring system (WTW, Germany) and an OxiTop®OC 110 controller were employed. On the other hand, twelve Velp® head sensors with inductive stirring systems from Velp® were used. Both devices were equipped with manometric sensors, but there was a significant difference in the stirring speed of each system. In the case of OxiTop, the stirring speed was 440 rpm, whereas in the Velp system, it was 60 rpm. Independently of the equipment used as bioreactors, amber glass bottles of 500 mL were employed, and a trap of sodium hydroxide (NaOH) was incorporated as a CO_2_ captor.

### Batches

A total of five trials were conducted separately. Each trial was composed of controls (with test medium and inoculum but no plastic), reference material (with test medium, inoculum, and a reference material of known high biodegradability) and two test materials (with test medium, inoculum, and the corresponding test material), all of which were tested in triplicate. One-third of each flask was filled with 164 ml of medium, and the headspace was left in the flask to increase the availability of O_2_. The reference material and the analysed biopolymers were added at a concentration of 0.1 g L^−1^. The bottles were incubated in a Thermostatically controlled cabinet (WTW TS 700/4i) and kept closed throughout the test, so no additional oxygen was provided. The test materials evaluated in each of the trials, the test temperature, the devices, and the incubation time are shown in Table 3.

**Table 3:** Specification of the test materials and conditions used in each of the batch experiments

Trial	Equipment	Stirring speed (rpm)	Test materials		Temperature ( °C)	Duration (days)
1	OXITOP	440	PLA	PHB	20	28
	VELP	60				
2	OXITOP	440	PLA	PHB	35	28
	VELP	60				
3	OXITOP	440	PLA	PHB	20	28
	VELP	60				
4	OXITOP	440	BGREEN	PHB	20	28
	VELP	60				
5	OXITOP	440	BMAT	BBEIGE	20	28
	VELP	60				

After 28 days, the BOD_28_ (Eq. 1) and the degree of biodegradation (Eq. 2) were determined for each test material.

### Statistical analysis

Changes in the BOD over time were investigated via generalised additive models (GAMs) with the R function ‘gam’ from the r package ‘mgcv’ (Wood and Wood 2015). The best model was selected based on the Akaike information criterion (AIC), with temperature, equipment and type of material included as fixed factors, while the trial was considered a random effect to account for variability associated with the different experimental conditions. The assumptions of heteroscedasticity and normality of the residuals of the model were checked using the Shapiro–Wilk test for normality and the Levene’s test for homogeneity of variances. Two-way ANOVAs were conducted to determine statistically significant differences between the biodegradation ratios reached by the different polymers in each trial, followed by Tukey’s HSD test to identify specific group differences. All analyses and figures were performed via R version 4.2.3 (R Core Team 2023).

## Results and discussion

### Variability in biochemical oxygen demand (BOD) values

**Fig. 1:**
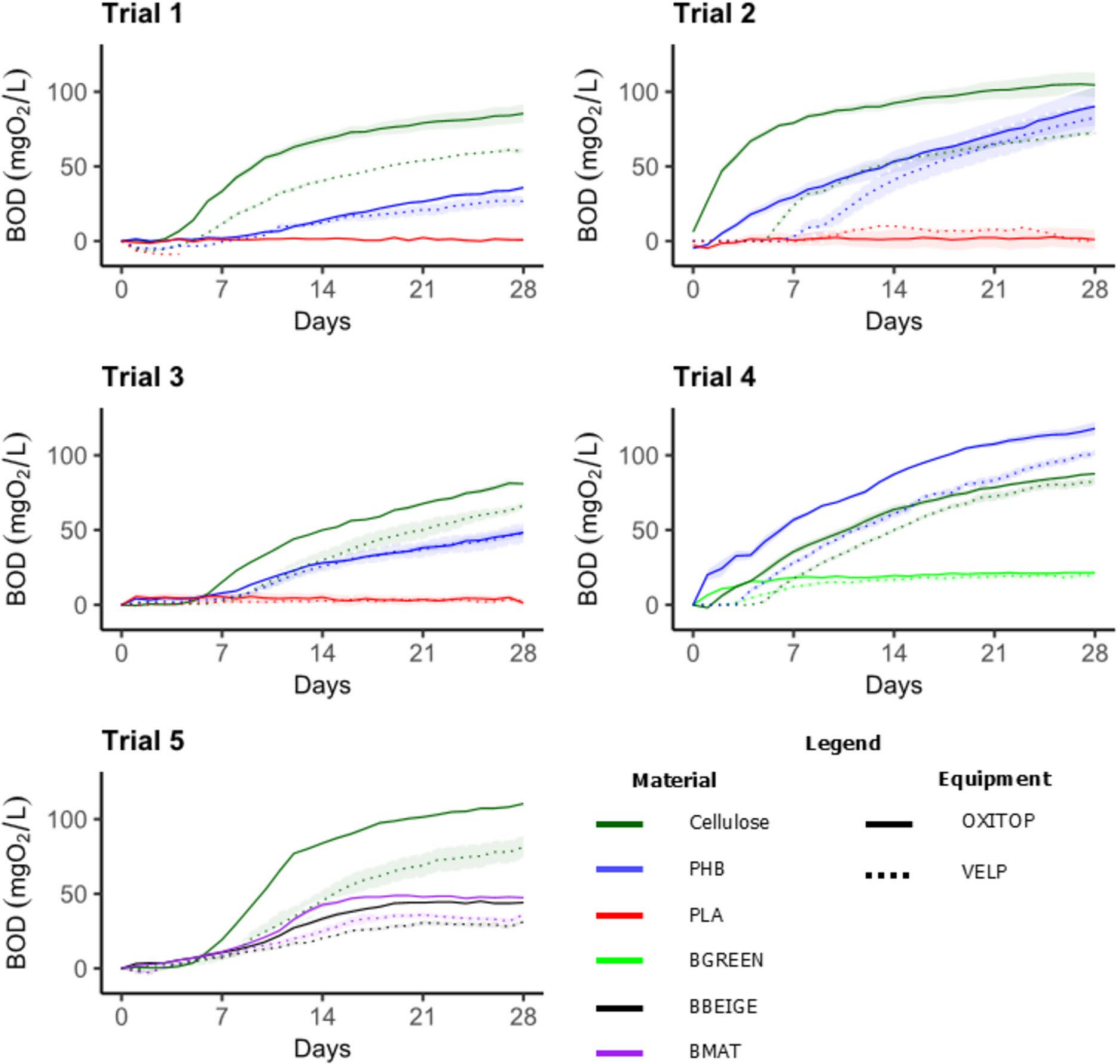
Daily variation in the biological oxygen demand (BOD) levels observed for each of the materials tested. The solid line corresponds to the values measured with the OXITOP equipment, and the dashed line corresponds to the VELP. Trials 1, 2 and 3: PHB and PLA test materials; Trial 4: BGREEN and PHB test materials; and Trial 5: BMAT and BBEIGE test materials. The data are mean values (N = 3), and the shadow represents the standard error

Figure 1 shows the BOD curves obtained for each of the tested materials after 28 days of incubation. First, the most notable finding was the statistically significant differences (p < 0.05) between the same materials (PHB and PLA) in different trials. Although the test medium was prepared according to the international standard protocol using identical reagents for all the experiments and the inoculum was consistently obtained from the same source, our results revealed the difficulty of replicating the probe at different times. This finding can be attributed to the high variability to which the bacterial populations used as inoculum are subjected, despite being sampled at the same WWTP for each trial. Guyard (2010) reported that in certified laboratories, the results of these tests have significant variability (> 20%) due to mainly the microbial population, an unacceptable limitation considering that these assessment tests should be replicable intra- and interlaboratory.

To confirm the activity of the inoculum and validate the methodology, the reference material (cellulose TLC) should achieve a minimum biodegradation degree of 60%. All trials carried out with the OXITOP devices met this threshold. However, in two out of five trials using VELP equipment, the cellulose content did not reach this limit (Table 4). All the experiments were developed within the valid range of the CFU and the concentration of suspended solids for the inoculum as specified by ISO 14851. Nevertheless, these requirements do not consider the differences attributable to the microbial community structure and diversity, which determines the quality of the inoculum and can potentially affect the results of these tests (Blok and Booy 1984; Goodhead et al. 2014). Šašinková et al. (2022) reported that differences in the microbial community of the inoculum can lead to complications in the reproducibility of tests. This is because some minority organisms may be crucial for the biodegradation of specific polymers.

**Table 4:**
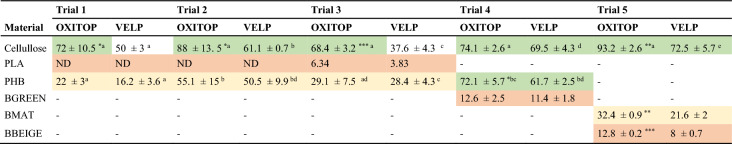
Percentage of biodegradation (D_t_) reached by the different materials after 28 days of incubation calculated as % ThOD

Each map is displayed by the relative intensity scale (0-1) where the color of each element is presented in strong bright (element-rich) and dark (element-deficient)

This variability was reflected in the cellulose results, with statistically significant differences (p < 0.05) in the BOD_28_ measurements between the different trials (Fig. 1). This variability, to which the positive control is also the subject in this type of experiment, is the best example to show the intralaboratory differences mentioned above. The BOD_28_ for PHB was also significantly different (p < 0.05) between trials (Fig. 1) with the same formulation and incubation conditions (Trials 1, 3 and 4). Similar results were observed when the curves for PLA were analysed. However, the differences between the values were not as pronounced as those observed for PHB, as the BOD_28_ values were lower. Nevertheless, there was still variation between the different experiments (Trials 1 and 3), and something remarkable was that we did not find an increase in the BOD measurements corresponding to PLA, not even when the temperature was increased to 35 °C (Trial 2). Although PLA has been shown to biodegrade at certain temperatures under controlled conditions such as composting (Stloukal et al. 2015), the same effect cannot be attributed to aqueous conditions, as demonstrated previously by Šašinková et al. (2022).

In addition, our results revealed statistically significant differences (p < 0.05) in the BOD_28_ values for the cellulose and each polymer when the results of the two devices (OXITOP and VELP) were compared in each test. Independent of the material, all the test materials reached higher BOD_28_ values in the OXITOP (solid line) than in the VELP equipment (dashed line) (Fig. 1). This finding reveals a previously unconsidered source of variability in international standards that govern this type of assessment test that may deeply impact the certification process depending on the equipment employed by the different laboratories. The variation in BOD or the variation in the consumption of oxygen observed depending on the instrument used can be attributed to the difference in stirring speed between the OXITOP (440 rpm) and the VELP (60 rpm). Mekaru (2020) reported the effect of agitation on the degradation of polystyrene. This author reported that the higher the agitation speed was, the greater the degree of mechanical abrasion to which the microplastics were subjected. This would increase the surface area of the particles, and as many authors have shown, the mineralisation rate of some polymers is correlated with the available surface area (César et al. 2009; Chinaglia et al. 2018). This could lead to different particle sizes between the two devices being smaller in the OXITOP; nevertheless, this is unlikely because, in our case, the tested materials were micronised. In our case, the higher BOD measurements in the OXITOP could be a consequence of the increased aeration caused by the faster agitation speed. This facilitates gas exchange between the headspace and the medium. These results contrast with those of previous studies (Prokešová 1962; Garcia et al. 2012), which reported that BOD was indirectly correlated with agitation and thus with the aeration of water.

### Is PHB a suitable reference material to assess biodegradation?

The variability shown by the BOD_28_ results was also reflected in the biodegradation percentage (Dt) of the tested materials after 28 days (Table 4). This outcome was expected because, according to ISO 14851, the biodegradation of plastics must be calculated from the BOD values. This variability is a serious problem considering that all products labelled biodegradable must meet several requirements set out in international standards (Iwata 2015; Filiciotto and Rothenberg 2021). Our findings demonstrated that performing the same test under similar conditions at various times can lead to different ultimate degrees of biodegradation for the same material owing to the instability of the inoculum or to the devices used in the tests.

The data represent the mean and the standard error for each of the trials (N = 3)

The background colour represents the biodegradability classification considering the same benchmarks used by the OECD for the classification of the persistence of chemicals in marine environments (OSPAR 2015). Red: Poorly biodegradable (0–20% ThOD). Yellow: Potentially biodegradable (20–60% ThOD) Green: Readily biodegradable (60–100% ThOD). ND: Biodegradation not detected

**Fig. 2:**
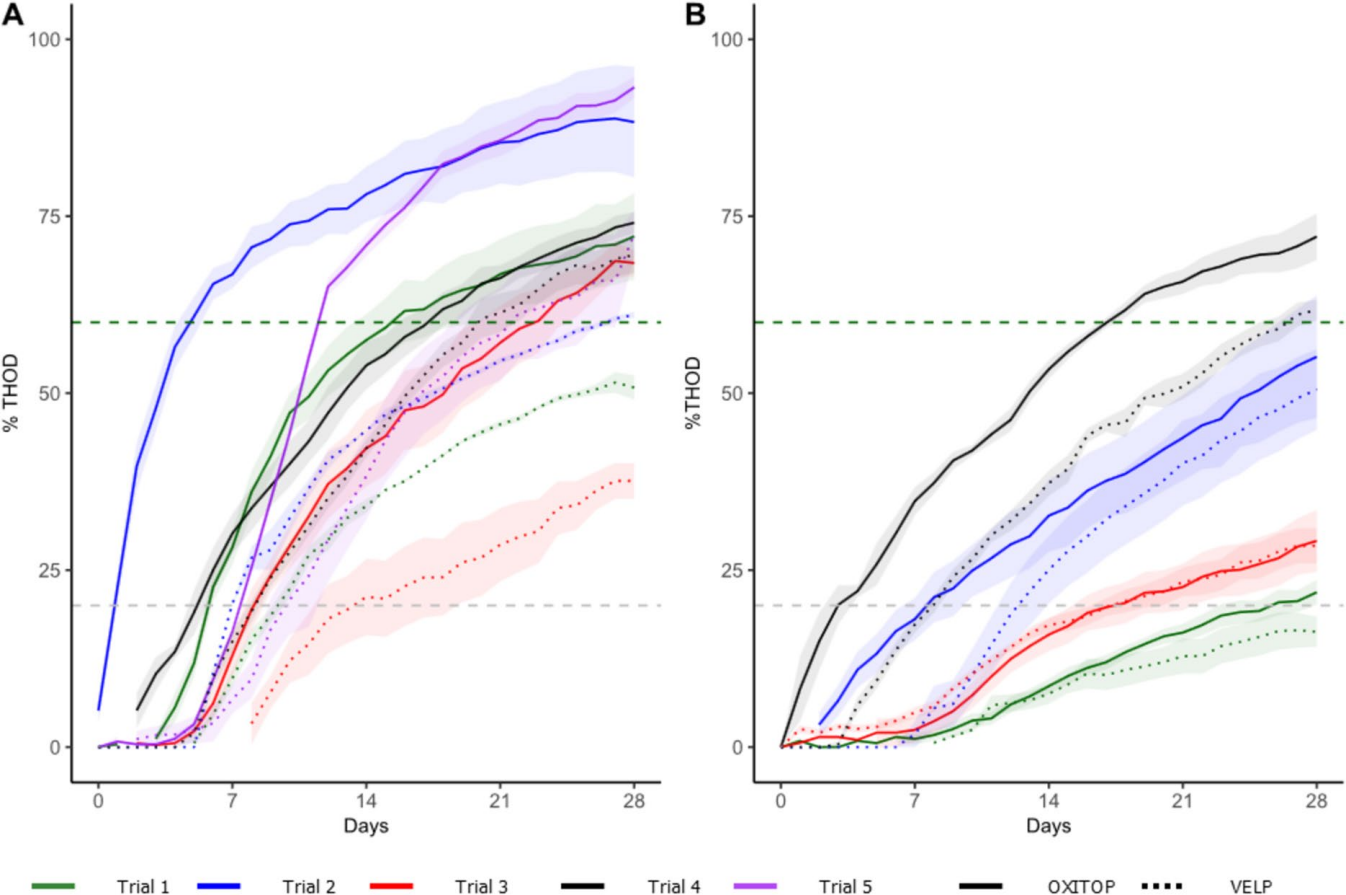
Different biodegradation curves of cellulose (**A**) and PHB (**B**) in each of the trials in which they were tested. Trial 2 was conducted at 35 °C, whereas the other trials were conducted at 20 °C. The horizontal dashed lines represent the biodegradability classification according to the OCDE. Poorly biodegradable (0–20% ThOD). Potentially biodegradable (20–60% ThOD). Readily biodegradable (60–100% ThOD). The data are mean values (N = 3), and the shadow represents the standard error

Previous studies have demonstrated the biodegradability of polyhydroxyalkanoates in different environments (Lee 2000; Kim and Rhee 2003; Alshehrei 2017), suggesting that polyhydroxyalkanoates are suitable reference materials for use as positive controls in biodegradation tests of polymers. However, our results (Fig. 2) revealed that although PHB is a biodegradable polymer and is not under discussion, the biodegradation of this compound shows high variability. These differences resulted in different respiration (BOD) curves (Fig. 1) and different degrees of ultimate biodegradation (Fig. 2). If PHB had been used as a positive control, the biodegradation assays could not be considered valid, as the reference material in all tests should have reached the 60% threshold set for acceptance of the results. Hence, the utilisation of PHB as a reference material in rapid biodegradation assessment tests for 28 days should be questioned, at least until the reproducibility of its biodegradation can be guaranteed, independent of the inoculum and equipment used. López-Ibáñez and Beiras (2022) previously used polyhydroxyalkanoates as reference materials to calculate biodegradation as a percentage of BOD28 compared with PHB (% C +). However, using this method in our case may lead to overestimation, as the compound used as a reference material did not reach a degree of biodegradation corresponding to readily biodegradable polymers. Although PHB is a biodegradable polymer, our results suggest that it may not be suitable as a reference material other than cellulose for freshwater biodegradation tests with an incubation time of 28 days. As shown in Fig. 2, in most of the trials, the test was stopped while the PHB was still in the biodegradation phase, so the test should be extended until the plateau phase has been reached.

### Degradation of two resins and three commercial bags

First, in the case of the resins, the highest degree of degradation was detected in the PHB samples (Table 4), while the level of PLA degradation comparable to that reported in previous studies, indicating the low biodegradability of this compound in aqueous media (Nazareth et al. 2019; Chamas et al. 2020). Moreover, consistent with the findings of Šašinková et al. (2022), increasing the temperature did not significantly increase PLA biodegradation. Temperature is the main limiting factor governing the biodegradation of PLA, as higher temperatures that exceeds the glass transition temperature (56–58 °C) are required to enhance the process, as it occurs during composting (Stloukal et al. 2015; Cosate de Andrade et al. 2016). According to our results PHB can be considered a readily biodegradable polymer in freshwater, although differences between trials and devices were detected, which could lead to its classification as a potentially biodegradable polymer following the adapted benchmarks of OSPAR (OSPAR 2015). However, PLA must be classified as a poorly biodegradable polymer since D_t_ never reached the 20% ThOD threshold, independently on the trial and equipment.

The biodegradation of the commercial bags was expected to be comparable to that obtained for PHB since, according to the manufacturers’ descriptions, they were composed of corn starch and a mixture of different biodegradable polymers. However, our findings showed, on the one hand, that the BMAT bag reached the highest degradability in both the OXITOP (35.69% ThOD) and VELP (33.69% ThOD) equipment. On the other hand, the results obtained for the BBEIGE and BGREEN bags were equal, ranging between 13 and 14%. According to the manufacturer’s description (Table 1), the BGREEN bag contains PLA, so thermophilic conditions are necessary for its degradation (Itävaara et al. 2002). López-Ibáñez and Beiras (2022) reported similar outcomes under marine conditions for commercial bags with the same composition as those used in our study. These authors attributed the biodegradation achieved to the mineralisation of the starch component of the bags. In these materials, which are composed of a mixture of polymers, the mineralisation of the most easily degradable compounds by the community of microorganisms present in the environment will occur first. The remaining compounds, whether biodegradable or not, will remain in the environment for more time in the same way as other organic waste in the absence of the specific conditions for their ultimate mineralisation (García-Depraect et al. 2022). Blending polymers to improve their mechanical properties could reduce the biodegradation potential of individual components (Narancic et al. 2020). This, coupled with the longer time required for the degradation of certain biopolymers such as PLA, can result in the accumulation of increasing amounts of these compounds in natural environments, which may represent a threat to biodiversity conservation (Awasthi et al. 2022). Indeed, some studies have reported the potential toxicity of bioplastics. Several degradation byproducts are released during the breakdown of some bioplastics which may leach into the environment (Kumar et al. 2024). These microplastics, if they persist in the environment, could adsorb toxic metals onto their surface, interact with biota, and potentially cause similar or even greater harm than microplastics derived from fossil fuel plastics. Additionally, various additives used to enhance the mechanical properties of bioplastics may leach into the environment, impacting both biota and the physicochemical characteristics of the surrounding medium (Ali et al. 2023).

Nowadays in the market there are available commercial plastic products that are certified as compostable but are also labelled with the term biodegradable when they cannot be biodegraded in other environments, at least according to the methodology established in ISO 14851. In addition, the high variability to which these assessment tests are subject, as has been shown in this study, may represent a major environmental threat. This variability transferred to industrial certification laboratories could lead to the classification of biodegradable materials that may not be biodegradable, at least not with the desired accuracy. This could led to the accumulation of bioplastic waste in natural environments that will require long term to biodegrade if this waste is not managed properly (Nazareth et al. 2022). Thus, the term biodegradable may be confusing to consumers in both the most appropriate application and the end-of-life disposal of the product.

## Conclusions

The method described in ISO 14851 is a suitable method for assessing the biodegradation of polymers in freshwater ecosystems; however, the source of the inoculum should be revised. Our results indicate that the use of activated sludge from a WWTP can cause large variability in BOD measurements and, consequently, in the ultimate degree of biodegradation. A limitation of this work has been the lack of characterisation of the microbiological communities of the inoculum. Further research is needed, testing different inoculum sources and characterising the composition of the different microbial communities through sequencing will help to understand which strains are responsible for degrading each polymer. Besides, due to population dynamics, the use of activated sludge as inoculum in standard tests makes it difficult or even impossible to carry out exact replicates of the same experiment. The search for alternative inoculum sources, more stable over time and with a known composition of microorganisms as for example commercial freeze-dried inoculum should be a priority line of research to improve these tests in the future. Additionally, the results of this study also revealed differences between the two different devices employed to carry out the tests, which may further maximise differences between laboratories. The biodegradation rates obtained with OXITOP ® equipment were higher rather than in the VELP. The identified difference between the two devices was the stirring speed but other potential differences have not been ruled out. The influence of this parameter on the biodegradation process should be addressed to understand the influence of this variable and further research is needed to assess other potential differences between equipment. Once the effect of this parameter is fully understood, it could lead to modifications in existing standards. Like the specification of parameters such as temperature, pH, or suspended solids concentration, a recommended stirring speed could be incorporated. It is therefore essential to investigate and refine existing standards to accurately determine the biodegradability of commercial products in freshwater environments. This will help prevent misinterpretation and misuse of these materials, reducing the risk of incorrectly labelling non-biodegradable compounds as biodegradable. Ultimately, this will ensure that they do not pose a threat to natural ecosystems.

## Data Availability

Data will be made avaliable under request to the corresponding author.
